# Chinese herbal formula (GCNY)-medicated serum alleviates peroxidation induced by H_2_O_2_ in human microglial cells

**DOI:** 10.3389/fnins.2022.990040

**Published:** 2022-09-14

**Authors:** Yong Chen, Baojiang Wang, Wing-Fu Lai, Yanjuan Chen, Rongbin Pan, Zhongsheng Tang, Dongzhou Liu

**Affiliations:** ^1^Division of Rheumatology and Research, Department of Geriatrics, The Second Clinical Medical College, Jinan University, Shenzhen People’s Hospital, Shenzhen, China; ^2^Institute of Maternal and Child Medicine, Affiliated Shenzhen Maternity and Child Healthcare Hospital, Southern Medical University, Shenzhen, China; ^3^Department of Urology, Zhejiang Provincial People’s Hospital, Hangzhou Medical College, Zhejiang, China; ^4^Department of Applied Biology and Chemical Technology, Hong Kong Polytechnic University, Hong Kong, Hong Kong SAR, China; ^5^Cancer Research Center, Jiangxi University of Traditional Chinese Medicine, Nanchang, China; ^6^Department of Anatomy, School of Basic Medicine, Guizhou University of Traditional Chinese Medicine, Guiyang, China

**Keywords:** traditional Chinese medicine, reactive oxygen species, inflammation, aging, oxidation, microglial cell

## Abstract

Traditional Chinese herbal medicine aiming at nourishing *yin* formed a distinctive school of thought in history to achieve anti-aging and longevity. In the formula Gancao nourishing *yin* (GCNY) decoction, all of the ingredients show antioxidant properties. However, in real clinical practice, extractions of herbs are rarely applied alone but are prescribed as the integrated formula. To investigate whether GCNY possesses anti-oxidation potential, we applied GCNY to treat rats to acquire medicated serum, which was then added on H_2_O_2_ (200 μM)-modeled human microglial cell line HMC-3 in comparison with its control serum. The results revealed that GCNY-medicated serum decreased reactive oxygen species (ROS) levels. Inflammatory cytokines such as pNF-κB p65 (ser536) and IL-6 were also decreased. Nrf2 and its pathway-related molecules, such as HO1, ABCC2, GLCM, ME1, NQO1, and TKT, were activated by H_2_O_2_ modeling while declined by treating with GCNY-medicated serum, which indicated attenuated oxidative stress of GCNY. Furthermore, mRNA-seq analysis showed 58 differential expressed genes (DEGs), which were enriched in pathways including antigen processing and presentation, longevity regulation, oxidative phosphorylation, and Parkinson’s disease progression. DEGs that were downregulated by H_2_O_2_ modeling but upregulated by GCNY treatment include CENPF, MKI67, PRR11, and TOP2A. Those targets were reported to be associated with the cell cycle and cell proliferation and belong to the category of growth factor genes. In conclusion, this study verified anti-oxidation effects of GCNY and indicated its promising application for cognitive degeneration and aging-related disorders.

## Introduction

Reactive oxygen species (ROS) levels and antioxidant defense mechanisms usually keep a dynamic homeostasis in a healthy body, which plays an important role in maintaining physiological functions (Brieger et al., 2012). However, when ROS is over-produced or the ability to eliminate ROS is weakened, oxidative stress occurs. This induces or aggravates a variety of diseases such as aging, heart disease, cancer, osteoarthritis, rheumatoid arthritis, and diabetes (Davalli et al., 2016; Climent et al., 2020) and brain tissue cognitive dysfunction caused by oxidative damage, such as Alzheimer’s disease and Parkinson’s disease (Singh et al., 2019; Agrawal and Jha, 2020). Stimuli of ROS lead to cell viability and secretory phenotype changes in microglial cells, which contributes to the complex responses of the neuroinflammatory process in the brain (Schmidt et al., 2021). It is also being recognized that the elimination of ROS is a major mechanism to treat neurodegenerative diseases (Ballance et al., 2019; Zhou et al., 2020).

Traditional Chinese medicine (TCM) shows promising advantages in treating degenerative diseases and for anti-aging purposes. Taking Zhu Danxi (AD. 1281–1358) for example, he was regarded as the representative of the theory of nourishing *yin*, he proposed the theory that the human body was inclined to suffer from a deficiency of *yin* while a surplus of *yang*, as shared TCM pathogenic mechanism for multiple age-related diseases (Gong et al., 2020). Gancao nourishing *yin* (GCNY) decoction is a Chinese herbal formula designed to tonify *yin*, while also promoting *yang* by body regulation. Previous studies validate its potential indications in age-related disorders such as constipation and ovarian cancer (Pan et al., 2022a), and the invention has been patented (ZL 2019 1 0843519.9 and ZL 2021 1 0515336.1) in China. Changes in metabolic profiles in the liver of hTNF-α transgenic mice were also observed (Pan et al., 2022b). It not only shows anti-inflammatory functions, but can also improve the health status (reflected by kidney Qi and blood of TCM theories) *via* the presence of herbal ingredients of licorice, ginseng, dandelion, Monk fruit, polygonatum, jujube, and ginger (Pan et al., 2022a). Based on these TCM theories and experimental results observed, we believe that the oxidation process, which shows profound interactions with metabolism and inflammation (Bekeschus et al., 2017), shall be proposed as the fundamental mechanism explaining how GCNY works.

Abundant studies on decreasing oxidative stress by chemical extracts of herbs present in GCNY were reported. Examples of these chemical extracts include licorice extracts (Kang et al., 2021) and ginsenosides from Ginseng (Jiang et al., 2021). However, it is important to notify that in clinical practice, TCM providers rarely apply a single extract, but always prescribes a formula of several herbal ingredients that may contain hundreds of chemicals. So, in this study, we try to explore that the GCNY formula affects the oxidative stress of HMC-3 cells, a human fetal brain-derived primary microglia. To conduct this *in vitro* study, GCNY-medicated serum and normal serum as controls obtained from rats were utilized to treat H_2_O_2_-modeled cells and their effects on oxidation are compared.

## Results

### Successful construct of oxidative stress cell model using H_2_O_2_

**FIGURE 1:**
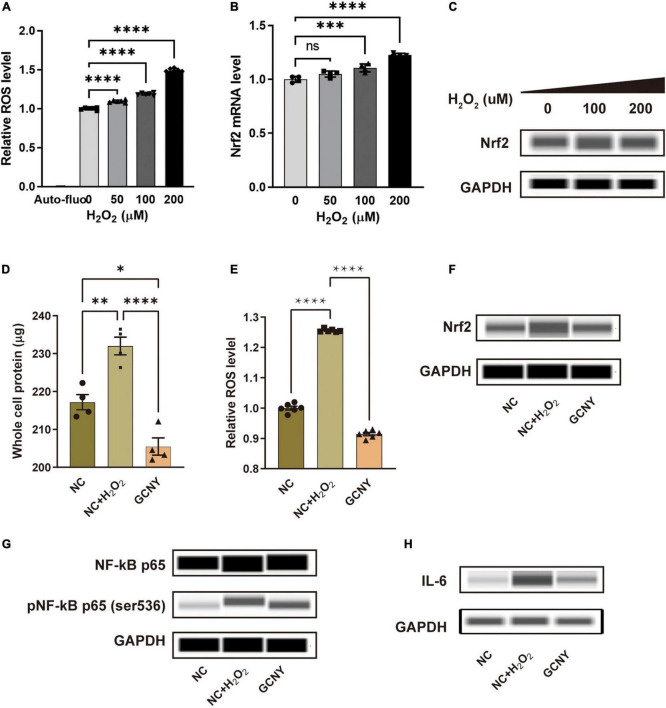
GCNY-medicated serum decreased Nrf2 levels and inflammatory cytokines of NF-κB and IL-6. **(A)** Intracellular relative ROS levels in HMC-3 cells treated with different concentrations (0, 50, 100, and 200 μm) of H_2_O_2_ for 4 h. Auto-fluo refers to the auto-fluorescence intensity value of HMC-3 cells. **(B,C)** The mRNA and protein levels of Nrf2 were expressed by HMC-3 treated with different concentrations of H_2_O_2_. **(D)** The significantly elevated whole protein level in NC + H_2_O_2_ group cells indicates an increased proliferation of HMC-3 cells compared to the NC group cells and **(E)** ROS levels were attenuated in cells treated by GCNY-medicated serum. **(F–H)** Through automated Western blot analysis, GCNY group cells expressed lower levels of Nrf2, NF-κs p65, pNF-κB p65 (ser536), and IL-6 compared with NC + H_2_O_2_ group cells. ns, no significance, *p < 0.05; ^**^p < 0.01; ^***^p < 0.001; ^****^p < 0.0001.

HMC-3 cells were treated with different concentrations of H_2_O_2_ (0, 50, 100, and 200 μm) for 4 h to induce an *in vitro* oxidative stress cell model. With the increase of H_2_O_2_ concentration, the level of intracellular ROS gradually increased (Figure 1A), suggesting a progressively aggravated state of oxidative stress. With the activation of the endogenous antioxidant system, the mRNA and protein levels of Nrf2 were examined, and both were upregulated (Figures 1B,C). Thus, the oxidative stress cell model was successfully constructed by adding H_2_O_2_ to HMC-3 cells, and concentration of H_2_O_2_ at 200 μm was chosen for the following experiments.

### GCNY-medicated serum decreased the reactive oxygen species level in HMC-3 cells

Increased intracellular oxidative tension leads to oxidative damage of cells, which can stimulate cell proliferation. However, the excessive proliferation of microglia is harmful to the interaction of neurons, thus affecting the cognitive function (Schmidt et al., 2021). The significantly elevated whole protein level in normal control serum-medicated cells added on H_2_O_2_ (NC + H_2_O_2_) indicated increased proliferation of HMC-3 cells compared to the NC cells (Figure 1D). It was found that the GCNY group did not cause excessive proliferation of HMC-3 cells. Compared with the NC + H_2_O_2_ group, the cell protein content was significantly decreased and slightly lower than that of the NC group in which H_2_O_2_ stimulation was absent (Figure 1D). The more direct evidence is that GCNY-medicated serum decreased ROS levels compared to its control group (NC + H_2_O_2_) cells (Figure 1E).

### GCNY-medicated serum decreased Nrf2 levels and inflammatory cytokines of NF-kB and IL-6

To control the ROS level and prevent its accumulation, the human body has formed a very complete antioxidant defense system, for example, the Nrf2 regulatory system. Nrf2 is the pivotal molecule of this system and, as an important redox-sensitive transcription factor, participates in maintaining redox homeostasis of cells by regulating endogenous antioxidant molecules. Nrf2 is activated and increased when oxidative stress occurs (Schmidlin et al., 2019). In this study, Nrf2 was activated in NC + H_2_O_2_ group cells. GCNY-medicated serum significantly lowers the Nrf2 expression in HMC-3 cells (Figure 1F), which indicated a decreased level of oxidation in cells of the GCNY group.

**FIGURE 2:**
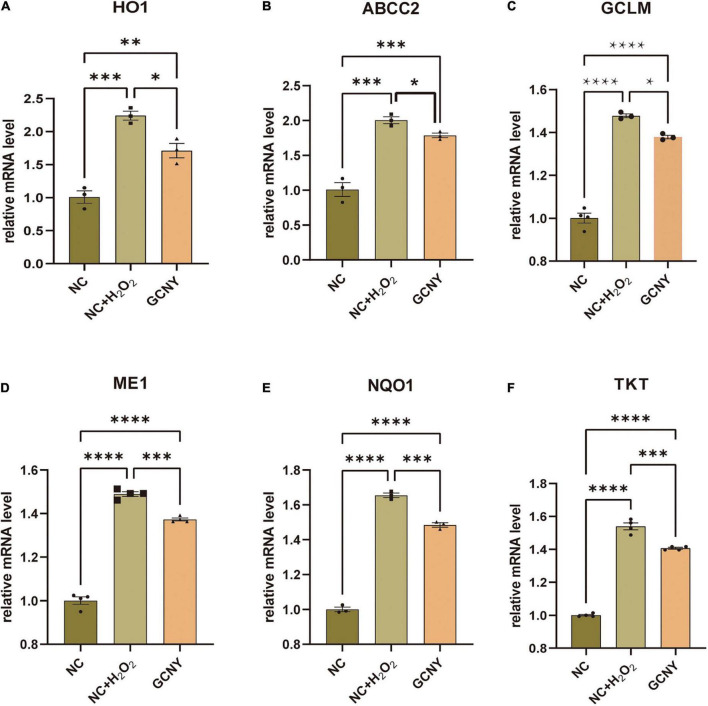
Increased oxidative tension in H_2_O_2_ modeling was reduced by GCNY-medicated serum. **(A–F)** Relative expression levels of Nrf2 pathway-related molecules, such as HO1, ABCC2, GLCM, ME1, NQO1, and TKT, were all elevated by H_2_O_2_ modeling in NC + H_2_O_2_ group cells, while lowered by adding GCNY-medicated serum. *p < 0.05; ^**^p < 0.01; ^***^p < 0.001; ^****^p < 0.0001.

The association between pro-inflammation and oxidation is the area we are interested in. NF-κB p65, pNF-κB p65 (ser536) (Ali et al., 2020), and IL-6 (Kaur et al., 2020) were proved to be effector cytokines and therapeutic targets in neurodegeneration and were all inhibited by GCNY-medicated serum (Figures 1G,H). Reduced oxidative tension by GCNY exhibited by lower expression levels of Nrf2 pathway-related molecules, such as HO1, ABCC2, GLCM, ME1, NQO1, and TKT (Figures 2A–F). The interaction between these targets and NF-κB p65, IL-6 is shown in Supplementary Figure 1.

### RNA-sequencing data analysis gross alternation by H_2_O_2_ modeling and GCNY treatment

To explore the impact of H_2_O_2_ modeling to HMC-3 cells and how GCNY-medicated serum affects the transcriptome of H_2_O_2_-modeled cells, high-throughput mRNA-sequencing analysis was performed in NC, NC + H_2_O_2,_ and GCNY groups of cells.

#### Quality control

**FIGURE 3:**
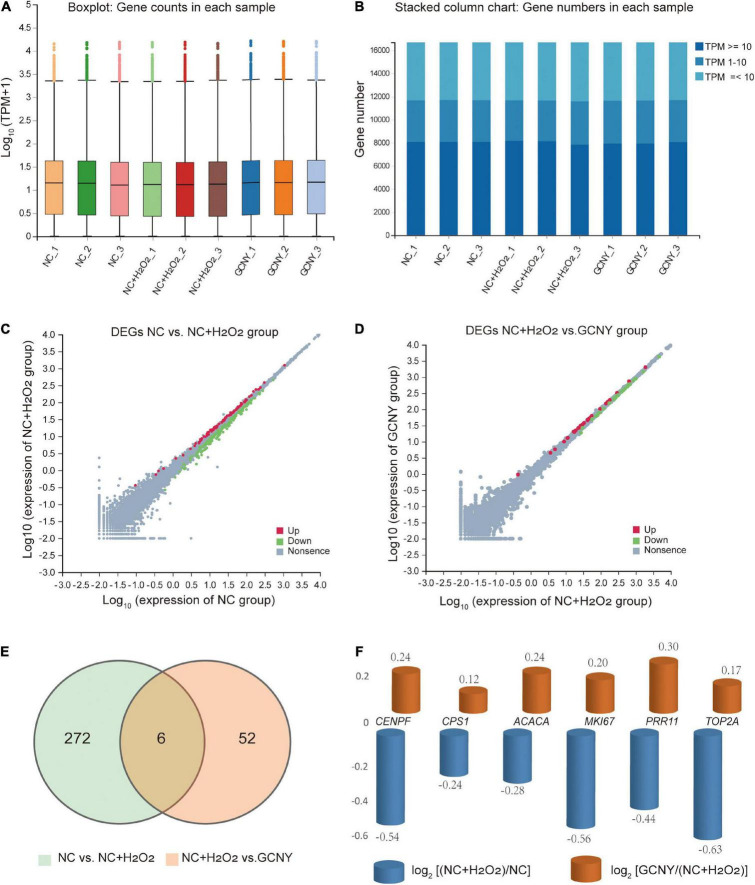
Analysis of RNA-sequencing data screened out gross alternation by H_2_O_2_ modeling and GCNY treatment. **(A,B)** Boxplot and stacked column chart represent expression uniformity of all inspected samples. **(C)** DEGs of NC vs. NC + H_2_O_2_ group cells. **(D)** DEGs of NC + H2O2 vs. GCNY group cells. **(E,F)** Six DEGs were downregulated in NC + H_2_O_2_ group cells by H_2_O_2_ modeling, but up-regulated in GCNY group cells.

In this project, a total of 9 samples from the 3 groups were measured using the DNBSEQ platform. Each sample produced an average of 6.65 G data. The average alignment rate of the sample alignment genome was 93.62%, and the average alignment rate of the aligned gene set was 74.60%. A total of 16,686 genes were detected. The boxplot shows the distribution of gene expression levels of all samples in each group, and the degree of dispersion of the expression data distribution can be observed (Figure 3A). To more intuitively show the number of genes in each sample in different Transcripts Per Million (TPM) intervals, we counted the number of genes in three cases of TPM (TPM ≤ 1, TPM 1-10, TPM ≥ 10) (Figure 3B). Either of the distributions indicated RNA expression uniformity of the inspected samples.

#### Differential expressed genes were observed after H_2_O_2_ modeling and GCNY treatment

Through H_2_O_2_ modeling, 89 mRNAs were upregulated whereas 189 were downregulated (Figure 3C and Supplementary File 2). Compared to NC *+* H_2_O_2_ group cells, GCNY-medicated serum upregulated 29 DEGs and downregulated 29 DEGs (Figure 3D and Supplementary File 3). Among the DEGs acquired, 6 mRNAs that were downregulated by H_2_O_2_ modeling were upregulated through the treatment of GCNY-medicated serum. They are CENPF, CPS1, ACACA, MKI67, PRR11, and TOP2A (Figures 3E,F). All of which were reported to be associated with the cell cycle and cell proliferation and belong to the category of growth genes. The interaction between these targets is shown in Supplementary Figure 2.

**FIGURE 4:**
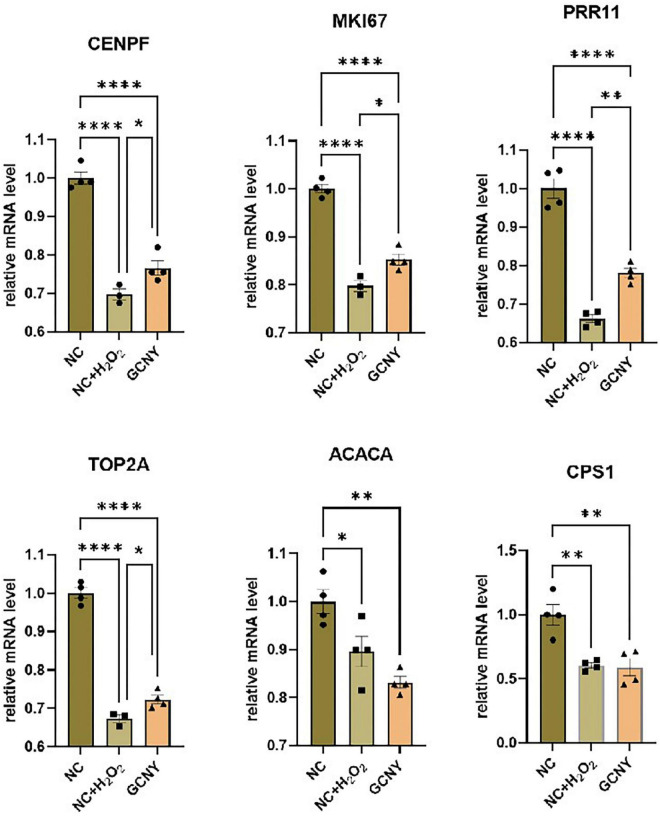
Validation the expression levels of DEGs through qRT-PCR assay. The selected DEGs are screened out by RNA-seq that downregulated by H_2_O_2_ modeling, while upregulated by GCNY-medicated serum. *p < 0.05; ^**^p < 0.01; ^****^p < 0.0001.

To further validate the expression of the above six DEGs, we detected their expression through qRT-PCR method. The expression changes of CENPF, MKI67, PRR11, and TOP2A were identical to the result of RNA-seq, except ACACA and CPS1, which failed to show significant changes between NC + H_2_O_2_ and GCNY group cells, and were still downregulated in NC + H_2_O_2_ group cells compared with NC group cells (Figure 4).

#### Representative differential expressed genes screened out by RNA-sequencing

**FIGURE 5:**
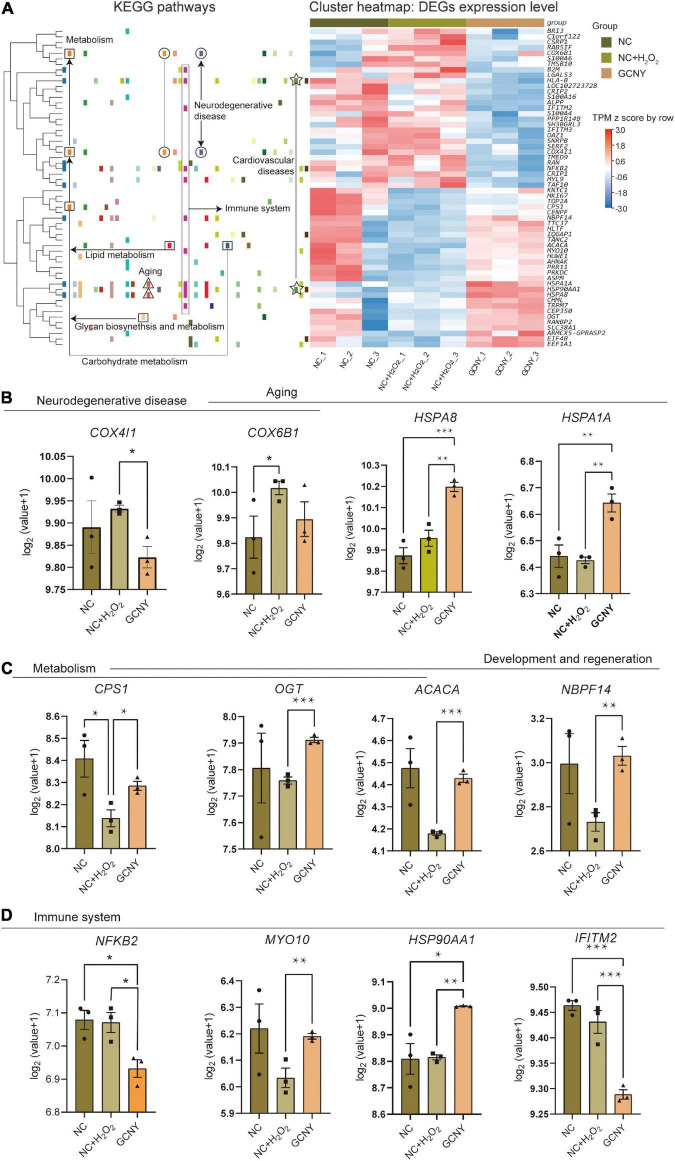
Representative DEGs screened out by RNA-sequencing. **(A)** Heatmap of 58 DEGs that resulted from GCNY-medicated serum treatment and their expression in NC, NC + H_2_O_2,_ and GCNY group cells. Representative DEGs enriched in pathways of **(B)** neurodegenerative disease and aging, **(C)** metabolism, and **(D)** development/regeneration and immune system. *p < 0.05; **p < 0.01; ***p < 0.001; ****p < 0.0001.

By Kyoto Encyclopedia of Genes and Genomes (KEGG) enrichment of 58 DEGs (29 upregulated and 29 downregulated) resulted from GCNY-medicated serum treated cells, they were assigned to various bio-activity pathways including those related to neurodegenerative diseases, cardiovascular diseases, immune system metabolism, and aging (Figure 5A). For example, COX4I1 and COX6B1 enriched in the neurodegenerative disease pathway are two subunits of cytochrome c oxidase, are an integral part of the mitochondrial machinery needed for ATP production in mammalian cells (Pajuelo et al., 2020), and are found to be overexpressed when neuronal cells are injured (Yang et al., 2019). Both of which were raised under H_2_O_2_ modeling while back to the normal level under GCNY treatment (Figure 5B). HSPA1A and HSPA8 are members of the heat shock protein 70 family, which contributes to the neuroprotection and proteostasis (Zinkie et al., 2013), which are the mechanisms usually participated in aging (Fernandez-Fernandez et al., 2017). Both of HSPA1A and HSPA8 were upregulated in GCNY group cells (Figure 5B).

The DEGs that participate in the pathways of metabolism and development/regeneration include CPS1, OGT, ACACA, and NBPF14. They were of different degrees downregulated by H_2_O_2_ modeling while upregulated by GCNY (Figure 5C). ACACA catalyzes the ATP-dependent carboxylation of acetyl-CoA, a rate-limiting step in fatty acid biosynthesis (Hunkeler et al., 2018). By far, not much function has been revealed on NBPF14. The mitochondrial enzyme encoded by CPS1 catalyzes the synthesis of carbamoyl phosphate from ammonia and bicarbonate, which plays an important role in removing an excess ammonia from the cell, and is widely studied in proteomic-based research; CPS1 was upregulated by an effective treatment in insulin resistance in mice (Ham et al., 2021). A recent study on OGT was remodeled and showed enhanced enzymatic activity in response to cellular oxidative stress (Martinez et al., 2021).

**NF-κB**2, which is a subunit of the transcription factor complex nuclear factor-kappa-B (**NF-κB**) and is usually activated by oxidative stress, was lower in its expression in GCNY-treated cells. The result is in accordance with the findings in the protein level above (Figure 1D). Similarly, IFITM2, which induces the innate immune system, was also downregulated (Figure 5B). Although MYO10 and HSP90AA1 are inflammations and cell growth factors, they are essential for brain development and cognitive maintenance (Yu et al., 2015; Xiong et al., 2021) and were upregulated by GCNY treatment (Figure 5D).

#### Pathways of differential expressed genes involved

**FIGURE 6:**
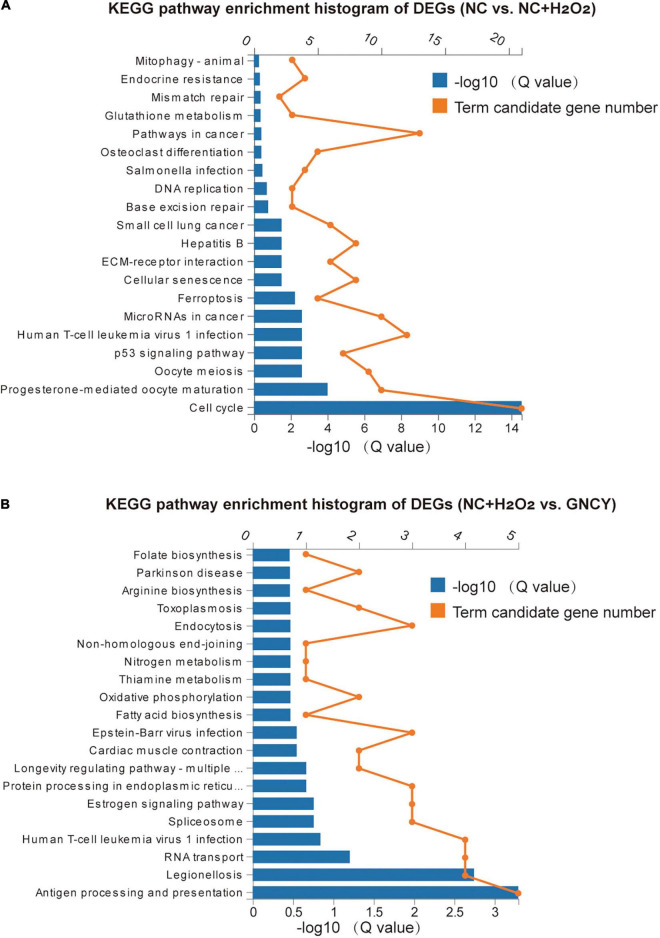
KEGG pathways of DEGs by H_2_O_2_ modeling and GCNY-medicated serum treatment. **(A)** Top 20 KEGG pathways enriched for the 278 DEGs resulted from H_2_O_2_ modeling. **(B)** Top 20 KEGG pathways enriched for the 58 DEGs resulted from NC + H_2_O_2_ vs. GCNY group cells.

For the 278 DEGs resulting from H_2_O_2_ modeling, they were enriched in KEGG pathways such as cell cycle, progesterone-mediated oocyte maturation, p53 signaling pathway, cellular senescence, ferroptosis, and pathways in cancer (Figure 6A). The Gene Ontology (GO) enrichment biological process of the DEGs includes cell cycle and division, chromosome segregation, regulation of cytokines (Supplementary Figure 3A), and molecular function including protein binding, microtuble binding, ATPase activity, and glutamate-cysteine ligase acclivity (Supplementary Figure 3B).

For the 58 DEGs resulting from NC **+** H_2_O_2_ vs. GCNY group cells, they were enriched in KEGG pathways including antigen processing and presentation, legionellosis, longevity regulating pathway, oxidative phosphorylation, thiamine and nitrogen metabolism, and Parkinson’s disease (Figure 6B). GO enrichment biological process includes protein refolding, innate immune response, and response to interferon, etc., (Supplementary Figure 4A) and includes molecular function of RNA binding, protein binding, and cytochrome c oxidase activity, etc. (Supplementary Figure 4B).

## Discussion

As long as life does not stop, oxygen is vital to keep oxidation reactions in the body all the time for all the organs, tissues, and cells to proceed with all necessary biological behavior. In addition, along with the oxidation reaction, degeneration and aging are inevitable. Moreover, prolonged or persistent exposure to non-physiological levels of oxidation stress and ROS has been proved to be associated with a variety of diseases, especially neurodegenerative diseases (Knaus, 2021; Zhou et al., 2022). Microglia are resident macrophages of the brain contributing to the innate immune response in the central nervous system. They could become activated by danger signals such as H_2_O_2_ (Schmidt et al., 2021). The activation process may accelerate pro-inflammatory responses with potential exacerbation of brain injury and can be served as a key player in neurodegeneration and also psychiatric disorders (Rahimian et al., 2021). In the current research, GCNY-medicated serum significantly decreased ROS levels in microglia HMC-3 cells and also inflammatory cytokines of NF-κB and IL-6.

Reactive oxygen species plays a controversial role in determining cell fate as excessive ROS triggers apoptosis signaling and leads to cell death; however, it can promote cellular transformation by the activation of signaling factors of extracellular-regulated kinase (ERK1/2), NF-κB, PI3K/AKT, and matrix metalloproteinase (MMP) to promote cellular proliferation (Sajadimajd and Khazaei, 2018). In our results, the increased cell protein levels of the H_2_O_2_-induced cells in culture plates indicated the activated cell proliferation behavior, but is restrained by GCNY-medicated serum. Coordinated induction of cytoprotective gene transcription through the antioxidant response element (ARE) is essential for cellular protection against oxidative stress and related disorders. Nrf2 is a cellular sensor of oxidative and electrophilic stress. Also, the main mediator of cellular adaptation to redox stress belongs to ARE members (Niture et al., 2014). Activation of Nrf2 protects cells from apoptosis and induce proliferation, metastasis, and chemoresistance (Sajadimajd and Khazaei, 2018). HO1 is one of the genes regulated through Nrf2 with the function of degrading heme and generating the antioxidant molecules (Loboda et al., 2016), and in H_2_O_2_ concentration alike, our study was also reported being elevated (Ma et al., 2016). Thus, the increased Nrf2 expression along with its pathway-related companions such as HO1, ABCC2, GLCM, ME1, NQO1, and TKT was activated and somewhat increased by ROS activation, and it is reasonable to interpret these targets that are not promoted by GCNY-medicated serum but is adaptively declined due to reduced ROS levels.

Our experimental results indicate the influences especially anti-oxidation of GCNY, but it is unlikely that GCNY directly regulates one or several targets as a chemical done. For example, Nrf2 is rather than promoted but declined synergistically to its multiple signaling pathway-related molecules. Another reason is hundreds of orally absorbable ingredients and the corresponding targets identified in the formula using the network pharmacology approach (Pan et al., 2022a). Much evidence believes that indirect and overall body regulation to the targets of a disease is a more reasonable explanation to how TCM works, and as being absorbed and circulated *in vivo*, the medicated serum contains the impact of a herb formula within the serum that provides accessibility for GCNY to verify its effects in vitro. Thus, we applied mRNA-sequencing to figure out how GCNY-medicated serum acts on HMC-3 cells based on the overall genotype changes. The results turned out that 58 DEGs were identified, among which 6 mRNAs (CENPF, CPS1, ACACA, MKI67, PRR11, and TOP2A) were downregulated by H_2_O_2_ modeling but upregulated in GCNY group of cells. They are oncogenes, which in another word are growth factor genes (Kontomanolis et al., 2020). The activation of growth genes is the basis for cell viability, which is the feature of young cells. However, as stated above, the living cell protein level treated by GCNY-medicated serum was not increased. We believe that this phenomenon is due to multiple targets GCNY being affected, and also, the current bioinformatics understanding to genes is far from sufficient. Apart from the limitations of current technologies, the screened targets did indicate a growth potential of the cells treated by GCNY-medicated serum. Interestingly, the decline in NF-κB2 was screened out, which is in accordance with automated Western blot results of NF-κB.

Kyoto Encyclopedia of Genes and Genomes pathway classification for the DEGs indicated that neurodegeneration, cardiovascular diseases, aging, immune system, and metabolism pathways are involved by H_2_O_2_ modeling and GCNY treatment. GO enrichment analysis shows that protein folding and binding are significantly related. To date, it has been recognized that protein homeostasis (proteostasis) is an essential pillar for correct cellular function. Impairments in proteostasis are encountered in aging and age-related diseases. In particular, heat shock protein 70 (Hsp70) has an essential role in protein folding, disaggregation, and degradation. Hsp70 provides a physical platform for the binding of client proteins, other chaperones, and cochaperones (Fernandez-Fernandez and Valpuesta, 2018). In this study, both HSPA8 and HSPA1A were upregulated by GCNY-medicated serum and thus might provide a protein homeostasis mechanism for interpreting the effects.

In addition, medicated serum is currently a popular and recognized protocol to conduct *in vitro* study on TCM formula. Different to chemical drugs or a single extract from herbal medicine, the traditional formula such as GCNY is likely to possess hundreds of chemical composition with oral bioavailability (OB) ≥30% and drug-like properties (DL) ≥0.18 (Pan et al., 2022a). Compositions of the formula digested and absorbed by rats, then interacted with body to regulate neuro-endocrine immune system (NEI), and then applied to the cells. Thus, medicated serum mimics highly how medicine influences the changes to the body *in vivo*. However, whether this proposed NEI changes also eventually occurred in brain tissue needs further *in vivo* validation.

In conclusion, *via* current research, we are confident with anti-oxidation effect of GCNY. Certain evidence for developing this TCM formula to alleviate over-oxidation-related diseases such as aging, atherosclerosis, and Alzheimer’s disease was provided. Omics analysis provides evidence of the effects from a holistic view and demonstrates potential working mechanisms. However, it needs in-depth research to uncover complicated interactions between the ingredients and molecular targets in the future.

## Materials and methods

### Preparation of medicated serum

The GCNY-medicated serum and its control serum were stored in nitrogen canister and applied to this research after rewarming in room temperature. The detailed serum preparation method was shown in our previously published paper (Pan et al., 2022a). Briefly, SPF grade, healthy SD male rats purchased from Animal Experimental Center of Southern Medical University [production license number: SCXK (Yue) 2016-0041] were assigned into 2 groups to produce GCNY-medicated serum and its control serum. The GCNY group was treated with decoction boiled from Gancao (Glycyrrhiza uralensis Fisch.), Ginseng (Panax ginseng C. A. Meyer), Yuzhu (Polygonatum odoratum (Mill.) Druce), Luo han guo (Momordicae grosvenori), Pu gong ying (Taraxacum mongolicum Hand.-Mazz.), Gan jiang (dried ginger, Zingiber officinale Roscoe), and Da zhao (Ziziphus zizyphus) at ratio of 6:3:4:5:2:3:2. Concentrates of 0.95 g crude medicine per 100 g body weight of the rat were administrated intragastrically, two times a day (equivalent to 6.3 times the amount for a 60 kg adult human) continuously as intragastric administration for 7 days. The control group rats were similarly administered with the equivalent amount of normal saline. On day 7, rats were anesthetized by intramuscular injection of Zoletil (5 mg/100 g, Virbac, France), the blood samples were collected from abdominal aorta, and serum was acquired *via* centrifugation. Then, all rats were euthanized by hyperanesthesia.

### Cell culture and construction of the cellular model of oxidative stress

Human microglia cell HMC-3 was cultured in minimum essential medium (MEM) containing 10% FBS and 1 mM sodium pyruvate. Cells were seeded into 6-cm plates and grown at 37°C in a saturated humidity atmosphere of 5% CO_2_. When the confluency of cells became up to 80–90%, cells were then treated with different concentrations of H_2_O_2_ (0, 50, 100, and 200 μm) for 4 h to induce an *in vitro* oxidative stress cell model. After stimulation with H_2_O_2_, cells were collected to measure their intracellular ROS levels (method addressed in 4.4).

### Treatment to the HMC-3

On HMC-3 that reaches 90% confluence in the culture bottle, we divided cells into 2 sets of 6-well plates and changed media to fresh MEM supplemented with 10% GCNY-medicated serum and 10% normal saline-medicated serum (NC) for 24 h, respectively. Management of each group of cells is shown in Table 1, and the flow chart of the study is shown in Supplementary Figure 5.

**TABLE 1:** Management to GCNY group of cells and its controls.

Group	Normal serum	H_2_O_2_	GCNY-medicated serum
Normal serum intervened regular cells (NC)	+	−	−
Normal serum intervened model cells (NC + H_2_O_2_)	+	+	−
GCNY-medicated serum intervened model cells (GCNY)	−	+	+

### Measurement of reactive oxygen species

Reactive oxygen species was measured using the ROS-sensitive fluorescent probe 2’,7’-dichlorodihydrofluorescein diacetate (DCFH-DA) with ROS assay kit (cat. S0033, Beyotime, China). In brief, cells were harvested and incubated with 5 μm DCFH-DA for 20 min at 37°C. After that, cells were washed and resuspended in PBS and then immediately measured for cellular fluorescence level using a GloMax-Multi detection system (Promgea, United States).

### Real-time PCR assay

Total RNAs were extracted by the NucleoSpin RNA Plus kit (cat. no. 740984, MN, Germany). Quantitative real-time (qRT)-PCRs were prepared using transgene kit. Relative quantification was achieved by the 2-ΔΔCt method with GAPDH as the reference gene. The primer sequences used are shown in Supplementary Table 1.

### Automated western blot analysis

Cell protein was extracted using RIPA lysis buffer containing protease and phosphatase inhibitor cocktails (cat. no. 539134, Millipore and cat. no. 78420, Thermo Fisher Scientific), and the protein concentration was determined using the protein assay kit (cat. no. 23200, Thermo Fisher Scientific, United States). Separation of proteins was subjected to the WES system (ProteinSimple, United States) and 12–230 kDa separation modules were used. For target proteins detection, the following antibodies were used: anti-NF- kB p65 (Cell Signaling Technology, #6956), anti-phospho- NF- kB p65 (Ser536) (Cell Signaling Technology, #3033), anti-Nrf2 (Cell Signaling Technology, #12721), anti-IL-6 (Abcam, ab9324), and anti-GAPDH (Novus Biologicals, NB300-325).

### High-throughput mRNA-sequencing analysis

High-throughput RNA-sequencing was performed using cells of NC, NC + H_2_O_2,_ and GCNY groups as mentioned in 4.2.

#### Total RNAs extraction

Total RNAs were extracted from the cells of each group using TRIzol (Invitrogen, Carlsbad, CA, United States) according to the manual instruction. About 60 mg of cells was grounded into powder by liquid nitrogen in a 2-ml tube, followed by being homogenized for 2 min and rested horizontally for 5 min. The mix was centrifuged for 5 min at 12,000 × *g* at 4°C. Then, the supernatant was transferred into a new EP tube with 0.3 ml of chloroform/isoamyl alcohol (24:1). The mix was shacked vigorously for 15 s and then centrifuged at 12,000 × *g* for 10 min at 4°C. After centrifugation, the upper aqueous phase where RNA remained was transferred into a new tube with equal volume of supernatant of isopropyl alcohol and then centrifuged at 13,600 rpm for 20 min at 4°C. After deserting the supernatant, the RNA pellet was washed two times with 1 ml of 75% ethanol, and then, the mix was centrifuged at 13,600 rpm for 3 min at 4°C to collect residual ethanol, followed by the pellet air dry for 5–10 min in the biosafety cabinet. Finally, 25–100 μl of DEPC-treated water was added to dissolve the RNA. Subsequently, total RNAs were qualified and quantified using a NanoDrop and Agilent 2100 bioanalyzer (Thermo Fisher Scientific, MA, United States).

#### mRNA library construction

Oligo(dT)-attached magnetic beads were used to purify mRNA. Purified mRNA was fragmented into small pieces with fragment buffer. Then, first-strand cDNA was generated using random hexamer-primed reverse transcription, followed by a second-strand cDNA synthesis. Afterward, A-Tailing Mix and RNA Index Adapters were added by incubating to end repair. The cDNA fragments obtained from previous step were amplified by PCR, and products were purified by Ampure XP Beads and then dissolved in EB solution. The product was validated on the Agilent Technologies 2100 bioanalyzer for quality control. The double-stranded PCR products from previous step were heated, denatured, and circularized by the splint oligo sequence to get the final library. The single-strand circle DNA (ssCir DNA) was formatted as the final library. The final library was amplified with phi29 to make DNA nanoball (DNB), which had more than 300 copies of one molecular, DNBs were loaded into the patterned nanoarray, and single-end 50 bases reads were generated on BGIseq500 platform (BGI-Shenzhen, China).

#### RNA data process

The sequencing data were filtered with SOAPnuke (v1.5.2) (Xu et al., 2018) by (1) removing reads containing sequencing adapter; (2) removing reads whose low-quality base ratio (base quality less than or equal to 5) is more than 20%; and (3) removing reads whose unknown base (“N” base) ratio is more than 5%; afterward, clean reads were obtained and stored in FASTQ format. The clean reads were mapped to the reference genome using HISAT2 (v2.0.4) (Kim et al., 2019). Bowtie2 (v2.2.5) (Langmead and Salzberg, 2012) was applied to align the clean reads to the reference coding gene set, and then, expression level of gene was calculated by SEM (v1.2.12) (Li and Dewey, 2011). The heatmap was drawn by pheatmap (v1.0.8) according to the gene expression in different samples. Essentially, differential expression analysis was performed using the DESeq2 (v1.4.5) (Love et al., 2014) with Q value ≤ 0.05. To take insight into the change of phenotype, GO^1^ and KEGG^2^ enrichment analysis of annotated differently expressed gene was performed by Phyper^3^ based on hypergeometric test. The significant levels of terms and pathways were corrected by Q value with a rigorous threshold (Q value ≤ 0.05) by Bonferroni (Midway et al., 2020).

### Statistical analysis

Statistical analysis was performed using GraphPad Prism 9.0 software. All the data were given as mean ± SD. Differences between two groups were evaluated for statistical significance using Student’s *t*-test. One-way ANOVA with Tukey’s multiple comparisons test was used to evaluate the differences among three or more groups. *P-*value < 0.05 was considered as statistically significant.

## Data availability statement

The RNA-seq datasets in this study are available through the NCBI Gene Expression Omnibus (GEO) database, with accession code GSE210945 (https://www.ncbi.nlm.nih.gov/geo/query/acc.cgi?acc=GSE210945).

## Ethics statement

The animal experiment was approved by the Ethics Committee of Shenzhen People’s Hospital.

## Author contributions

YC and DL: concept and design. YC, W-FL, and RP: experiment performance. YC, YJC, and ZT: acquisition, analysis, and interpretation of data. YC, BW, and W-FL: drafting the manuscript. All authors contributed to the article and approved the submitted version.

## Footnotes

1
http://www.geneontology.org/

2
https://www.kegg.jp/

3
https://en.wikipedia.org/wiki/Hypergeometric_distribution
