# Supplementary material for: Chinese herbal formula (GCNY)-medicated serum alleviates peroxidation induced by H2O2 in human microglial cells
Source: Front Neurosci. 2022 Sep 14;16:990040. doi: 10.3389/fnins.2022.990040 (PMC9515651; doi:10.3389/fnins.2022.990040)
Supplement: Supplementary file 3 [file Data_Sheet_1.docx]

Supplementary Material

.
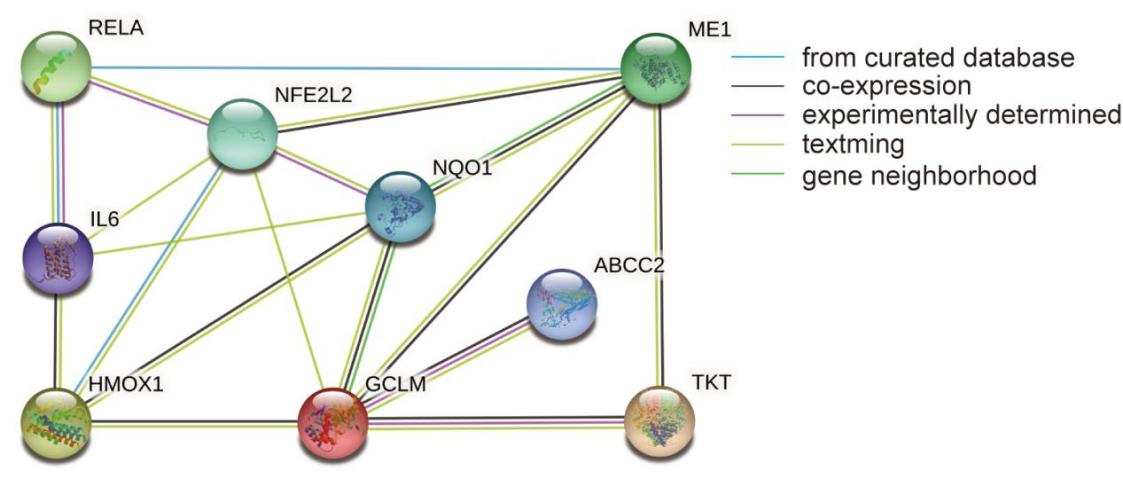


**Supplementary Figure 1.** PPI of selected targets of Nrf2 pathway. By imputing HO1 (preferred name HMOX1), CMOAT (preferred name ABCC2), GLCLR (preferred name GCLM), ME1, NQO1, TKT, NFKB3 (preferred name RELA), IL6 and NRF2 (preferred name NFE2L2) into String database (https://string-db.org/), we acquiring PPI of the above chosen targets.


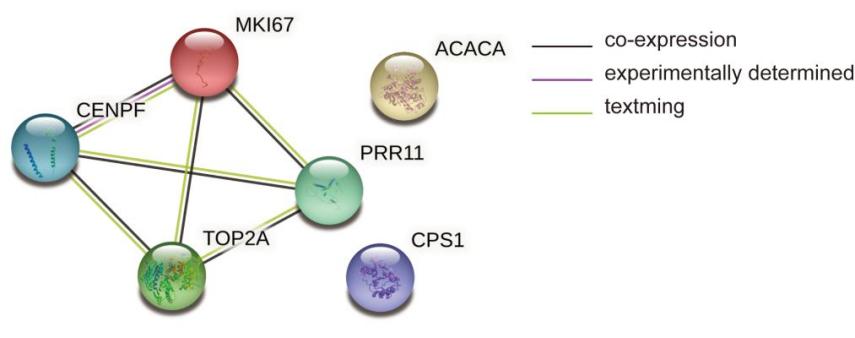


**Supplementary Figure 2.** PPI of targets down-regulated by H_2_O_2_ modelling but up-regulated through GCNY treatment. Performed by String database (https://string-db.org/)


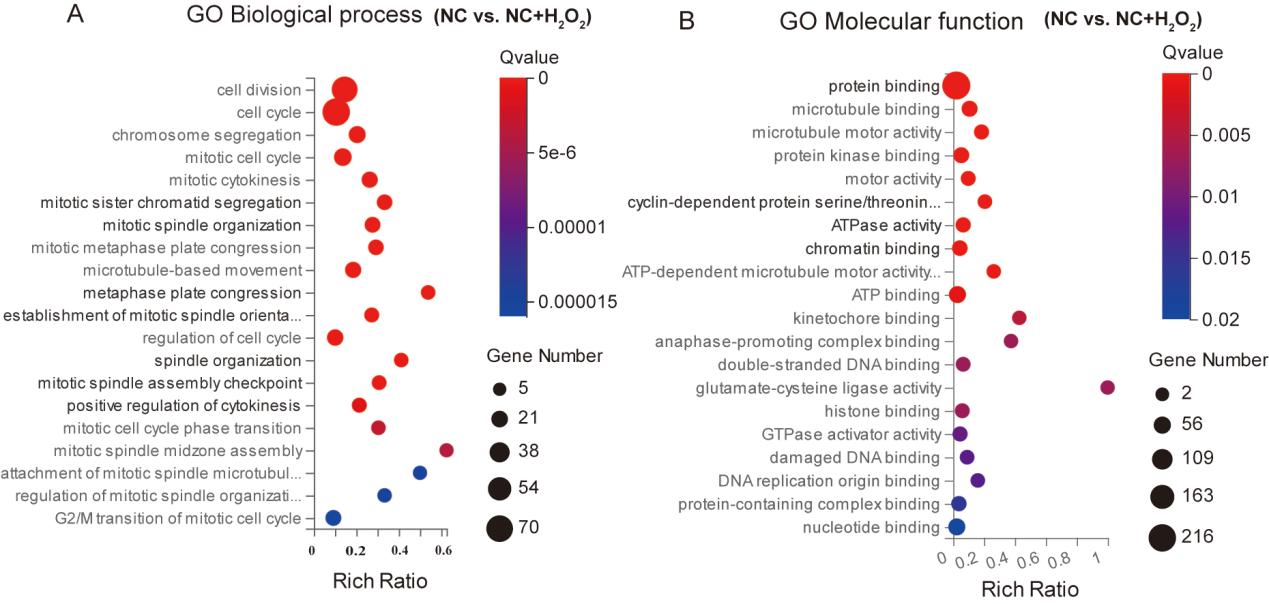


**Supplementary Figure 3.** GO enrichment of DEGs of NC vs. NC+H_2_O_2_ group cells.


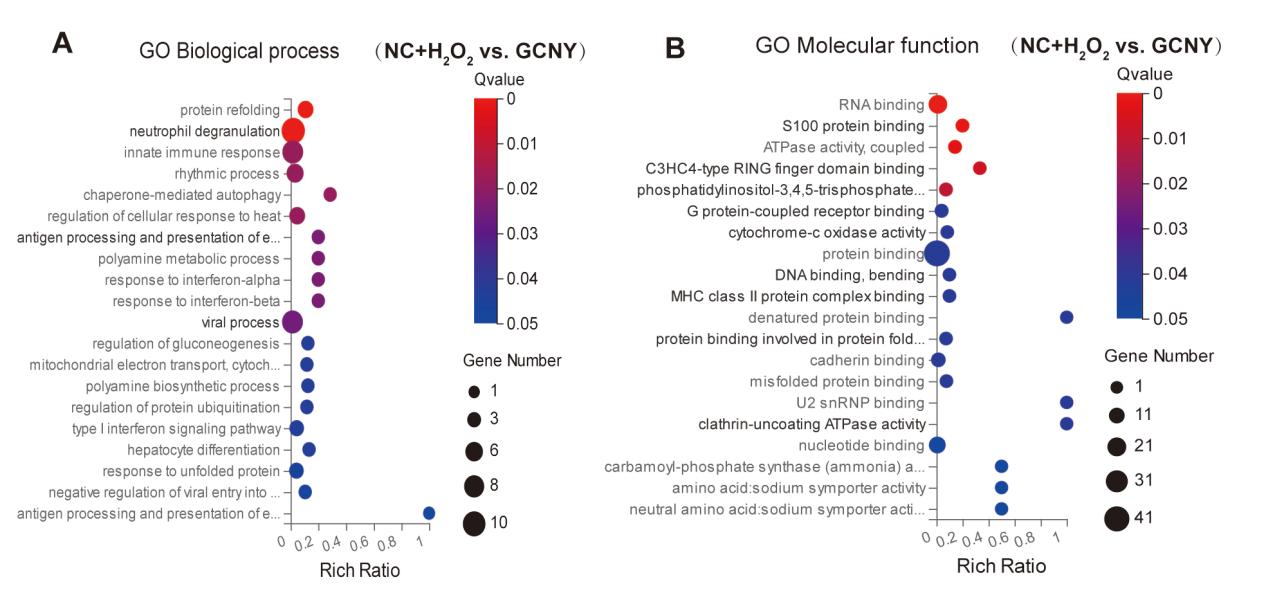


**Supplementary Figure 4.** GO enrichment of DEGs of NC+H_2_O_2_ vs. GCNY group cells.

**Supplementary Table 1.** The primer sequences used in the study

| **Genes** | **forward primer** | **reverse primer** |
| --- | --- | --- |
| HO1 | 5’-CTTTCAGAAGGGCCAGGTGA-3’ | 5’-GTAGACAGGGGCGAAGACTG-3’ |
| NQO1 | 5’- GGTTTGGAGTCCCTGCCATT-3’ | 5’- TTGCAGAGAGTACATGGAGCC-3’ |
| GCLM | 5’- GGGAACCTGCTGAACTGG-3’ | 5’- CTGGGTTGATTTGGGAACTC-3’ |
| ABCC2 | 5’- CTTGGGCTACCTATGGCTCC-3’ | 5’- ATCGAACAGCAGGGACTGTG-3’ |
| ME1 | 5’- CTGCCTGTCATTCTGGATGT-3’ | 5’- ACCTCTTACTCTTCTCTGCC-3’ |
| TKT | 5’- GCTGAACCTGAGGAAGATCA-3’ | 5’- TGTCGAAGTATTTGCCGGTG-3’ |
| CENPF | 5’- CGCCAGAACTGTACTCTCCG-3’ | 5’- GTAGGCAGCCCTTCTTTCCA-3’ |
| CPS1 | 5’- CTTTGGCCATCCATCCTCTG-3’ | 5’- TTGGCCATtGTGAGAATCTG-3’ |
| ACACA | 5’- AGATGTTTCGGCAGTCCCTG-3’ | 5’- ATGTGGACCAGCTGACCTTG-3’ |
| MKI67 | 5’- CGTCCCAGTGGAAGAGTTGT-3’ | 5’- CGACCCCGCTCCTTTTGATA-3’ |
| PRR11 | 5’-AAAGATGGACCCATGCAGATAAC-3’ | 5’- TGCTTTCGGCGATGGTATAAG-3’ |
| TOP2A | 5’- TTCTTGATATGCCCCTTTGG-3’ | 5’- GCTTCAACAGCCTCCAATTC-3’ |
| GAPDH | 5’- GTCAGCCGCATCTTCTTTTG-3’ | 5’- GCGCCCAATACGACCAAATC-3’ |


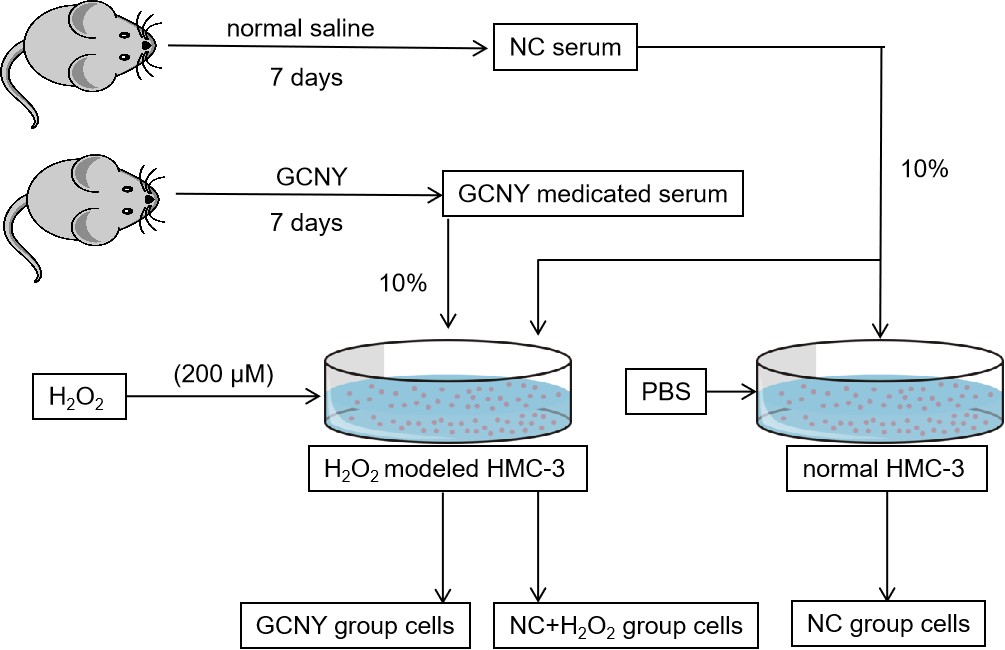


**Supplementary Figure 5**. Flow chart of preparing cells of NC, NC+H_2_O_2_ and GCNY groups.
